# Mechanisms of Diabetes-Induced Endothelial Cell Senescence: Role of Arginase 1

**DOI:** 10.3390/ijms19041215

**Published:** 2018-04-17

**Authors:** Esraa Shosha, Zhimin Xu, S. Priya Narayanan, Tahira Lemtalsi, Abdelrahman Y. Fouda, Modesto Rojas, Ji Xing, David Fulton, R. William Caldwell, Ruth B. Caldwell

**Affiliations:** 1 Vascular Biology Center, Medical College of Georgia, Augusta University, Augusta, GA 30912, USA; eshosha@augusta.edu (E.S.); zhxu@augusta.edu (Z.X.); pnarayanan@augusta.edu (S.P.N.); tlemtalsi@augusta.edu (T.L.); afouda@augusta.edu (A.Y.F.); mrojas@augusta.edu (M.R.); jxing8733@163.com (J.X.); dfulton@augusta.edu (D.F.); 2 Charlie Norwood VA Medical Center, Augusta, GA 30904, USA; 3 Department of Occupational Therapy, College of Allied Health Sciences, Augusta University, Augusta, GA 30912, USA; 4 Department of Pharmacology and Toxicology, Medical College of Georgia, Augusta University, Augusta, GA 30912, USA; wcaldwel@augusta.edu

**Keywords:** senescence, diabetes mellitus, diabetic retinopathy, arginase, retina, endothelial cells, hyperglycemia

## Abstract

We have recently found that diabetes-induced premature senescence of retinal endothelial cells is accompanied by NOX2-NADPH oxidase-induced increases in the ureohydrolase enzyme arginase 1 (A1). Here, we used genetic strategies to determine the specific involvement of A1 in diabetes-induced endothelial cell senescence. We used A1 knockout mice and wild type mice that were rendered diabetic with streptozotocin and retinal endothelial cells (ECs) exposed to high glucose or transduced with adenovirus to overexpress A1 for these experiments. ABH [2(S)-Amino-6-boronohexanoic acid] was used to inhibit arginase activity. We used Western blotting, immunolabeling, quantitative PCR, and senescence associated β-galactosidase (SA β-Gal) activity to evaluate senescence. Analyses of retinal tissue extracts from diabetic mice showed significant increases in mRNA expression of the senescence-related proteins p16^INK4a^, p21, and p53 when compared with non-diabetic mice. SA β-Gal activity and p16^INK4a^ immunoreactivity were also increased in retinal vessels from diabetic mice. A1 gene deletion or pharmacological inhibition protected against the induction of premature senescence. A1 overexpression or high glucose treatment increased SA β-Gal activity in cultured ECs. These results demonstrate that A1 is critically involved in diabetes-induced senescence of retinal ECs. Inhibition of arginase activity may therefore be an effective therapeutic strategy to alleviate diabetic retinopathy by preventing premature senescence.

## 1. Introduction

Recent studies have shown an association between diabetic retinopathy and premature senescence [1,2,3]. Senescence associated cytokines are increased in vitreous samples from patients with proliferative diabetic retinopathy [1]. Increased oxidative and nitrative stress has been linked to premature senescence in diabetic rat retinas [3]. These reports highlight the involvement of premature senescence in diabetic retinopathy. However, the underlying mechanisms are not clear.

We have previously reported an association between the diabetes-induced premature endothelial cell (EC) senescence and NOX2-NADPH oxidase-mediated decreases in nitric oxide synthase (NOS)-dependent nitric oxide (NO) bioavailability and increases in the expression/activity of the ureohydrolase enzyme arginase 1 [2]. Arginase exists in two isoforms: arginase 1 (A1) (the cytosolic isoform) is highly expressed in the liver, arginase 2 (A2) (the mitochondrial isoform) is highly expressed in the kidney. Both of the isoforms are expressed in other tissues, including the retina [4]. Arginase utilizes the semi-essential amino acid l-arginine as a substrate to produce l-ornithine and urea. l-arginine is also the substrate used by NOS to produce NO. Hence, under conditions of excessive arginase activity less l-arginine is available for NOS. When the l-arginine supply is limited NOS uses molecular oxygen as substrate to form superoxide, thereby decreasing NO formation, increasing oxidative stress, and causing vascular dysfunction. Excessive arginase activity has been shown to promote vascular dysfunction in a variety of diabetic complications, including diabetic retinopathy [4,5,6,7,8,9,10,11,12]. The present study was undertaken to investigate the specific involvement of arginase 1 in diabetes-induced premature retinal EC senescence.

## 2. Results

### 2.1. Diabetes-Induced Increases in Arginase 1 and Premature Cellular Senescence

**Figure 1:**
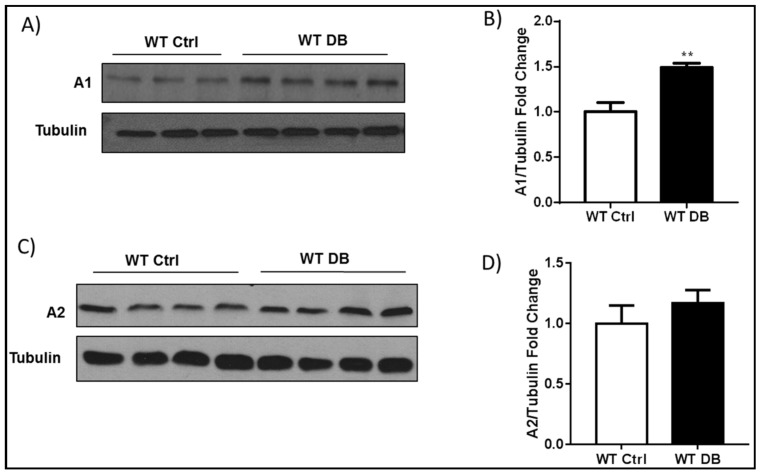
Diabetes induces increases in arginase 1 (A1) expression. (**A**) Western blot analysis and quantification (**B**) showing increased A1 expression in wild type (WT) diabetic retinas. ** *p* < 0.01 vs. WT Ctrl. *n* = 3–4; (**C**) Western blot analysis and quantification (**D**) show similar levels of A2 expression in WT diabetic mice and age matched controls. *p* = 0.4, *n* = 4.

Previous studies in models of diabetes and diabetic retinopathy have shown that increases in arginase 1 expression are associated with cell stress and vascular dysfunction [4,5]. Western blot analyses using whole retina tissue extracts confirmed a significant increase in arginase 1 protein levels in the diabetic retina as compared with age matched controls (Figure 1A,B). Levels of arginase 2 were not significantly altered (Figure 1C,D).

Analysis of isolated retinal vessels using a mouse cellular senescence PCR array showed significant increases in the expression of several senescence-associated genes in the vessels from diabetic mice as compared with the non-diabetic control mice (Supplementary Table S1). Levels of mRNA for the senescence pathway gene Cdkn1a (cyclin-dependent kinase inhibitor p21) were increased ~2.8-fold. The p21 protein represents a major target of p53 activity and is thus associated with linking DNA damage to cell cycle arrest [13]. Two genes that were involved in insulin signaling, Igfbp3 (insulin-like growth factor binding protein 3) and Igfbp7 (insulin-like growth factor binding protein 7) were increased by eight-fold and 1.6-fold, respectively. Igfbp3 has been shown to promote premature senescence of human umbilical vein endothelial cells (HUVEC) [14]. Igfbp7 has been found to reduce tumor growth by induction of senescence and apoptosis pathways [15].

**Figure 2:**
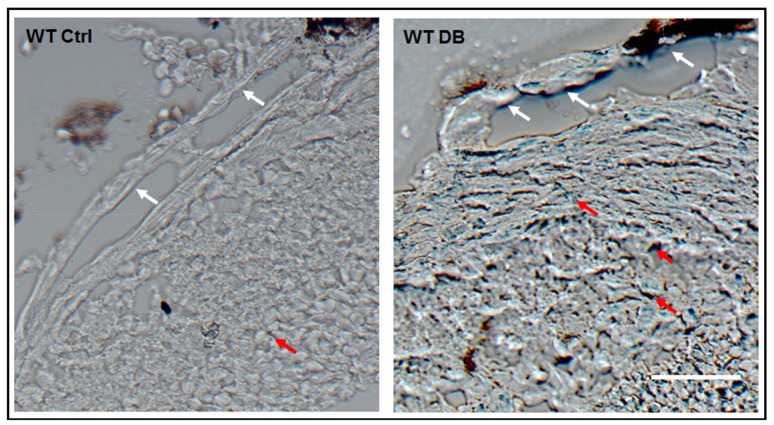
Diabetes induces senescence in retinal tissue. Senescence associated β-galactosidase (SA β-Gal) activity images of frozen retinal sections showing positive signal in cells of the large vessels (white arrows) as well as in the surrounding tissues (red arrows) of the central retina. Scale bar = 20 µM.

Previous studies have also demonstrated premature senescence in murine models of ischemic retinopathy, including diabetic retinopathy, by staining of retinal tissue sections using a colorimetric assay for senescence associated β-galactosidase (SA β-gal) activity [1,2,3]. β-galactosidase is a lysosomal enzyme that is active at a pH of 4 in normal cells. In senescent cells β-galactosidase is active at a pH of 6 and can be detected by adding the x-Gal substrate (5-Bromo-4-chloro-3-indolyl β-d-galactopyranoside), which forms a blue colored product [16]. Our analyses of retinal tissue sections using this method confirmed prominent increases in SA β-Gal activity in cells of the large vessels, as well as in the retinal parenchyma (Figure 2).

### 2.2. Suppression of Diabetes-Induced Increases in Senescence Markers by Inhibition of Arginase

**Figure 3:**
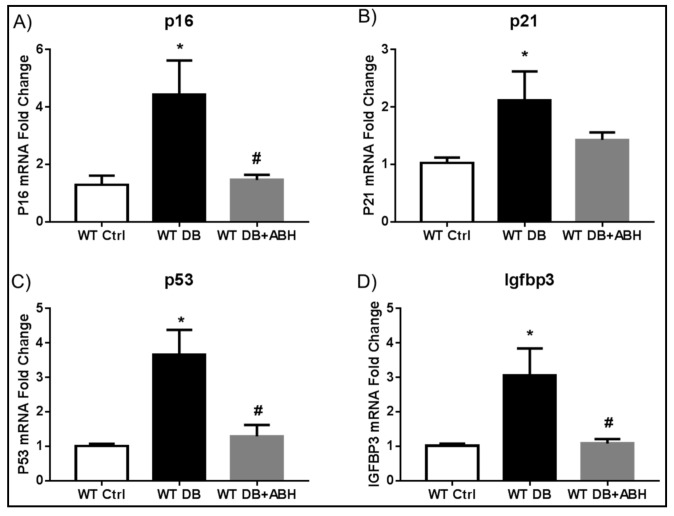
Arginase inhibition prevents diabetes-induced alterations in retinal senescence markers. qRT-PCR showing increased mRNA levels of p16 (**A**); p21 (**B**) p53 (**C**); and, Igfbp3 (**D**) in the diabetic retinas compared to age matched controls. 2(S)-Amino-6-boronohexanoic acid (ABH) treatment significantly blocked the diabetes-induced senescence. * *p* < 0.05 vs. WT Ctrl, # *p* < 0.05 vs. WT DB. *n* = 4–9.

Quantitative RT-PCR analysis of whole retinas from additional groups of diabetic mice confirmed significant increases in mRNA levels for p21 and Igfbp3 along with p16^INK4^ (cyclin-dependent kinase inhibitor 2A) and the tumor suppressor protein p53 as compared with non-diabetic control mice (Figure 3). These diabetes-induced alterations were completely blocked in diabetic mice that were treated with the arginase inhibitor ABH, demonstrating the involvement of arginase activity in this process. Additional studies of freshly isolated retinal vessels showed diabetes-induced increases in Igfbp3, as well as a trend towards increases in p16^INK4^ and p53. These alterations were abrogated by treatment of the diabetic mice with ABH (Supplementary Figure S1).

**Figure 4:**
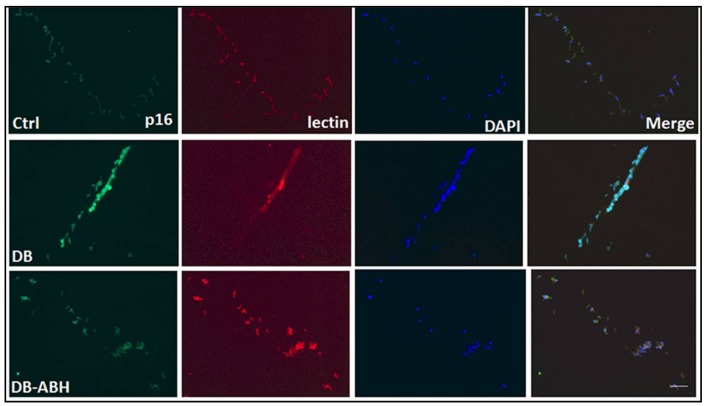
Arginase inhibition reduces p16 expression in isolated vessels. Immuno-labeling of isolated retinal vessels showing expression of the senescence protein p16 (green) in vascular endothelial cells which was co-localized with the endothelial marker (isolectin B4, red). Diabetic vessels showed increased p16 expression compared to controls. Arginase inhibition with ABH significantly reduced p16 expression. *n* = 3–4. Scale bar = 50 µM.

To further evaluate the involvement of arginase activity within the vascular endothelium in the diabetes-induced premature senescence, we assessed immunoreactivity for p16^INK4A^ in retinal vessels that were isolated from the control, diabetic, and ABH-treated diabetic mice. Immunofluorescence imaging showed that p16^INK4A^ was expressed in vascular endothelial cells, as demonstrated by its co-localization with isolectin B4 (Figure 4). Moreover, the immunoreactivity for p16^INK4A^ was increased in the vessels from the diabetic mice as compared with the non-diabetic controls and the diabetes-induced increase in p16^INK4A^ immunoreactivity was markedly blunted in the vessels from the diabetic mice that were treated with ABH.

**Figure 5:**
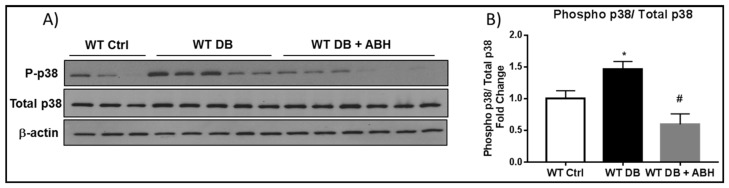
Diabetes induced- increases in phospho p38 is prevented by inhibiting arginase. (**A**) Western blot analysis and quantification (**B**) showing increased phosphorylation of p38 in WT diabetic retinal extracts compared to age matched controls. ABH treatment significantly blocked this increase. * *p* < 0.05 vs. WT Ctrl. # *p* < 0.001 vs. WT DB. *n* = 6–8.

The diabetes-induced increase in cellular senescence was accompanied by increases in cell stress, as shown by increased phosphorylation of p38 MAP kinase in whole retinal extracts when compared with the controls (Figure 5A,B). The increase in phosphorylated p38 MAP kinase was completely blocked by treatment of the mice with the arginase inhibitor ABH, indicating the involvement of arginase activity in the diabetes-induced cellular stress response.

**Figure 6:**
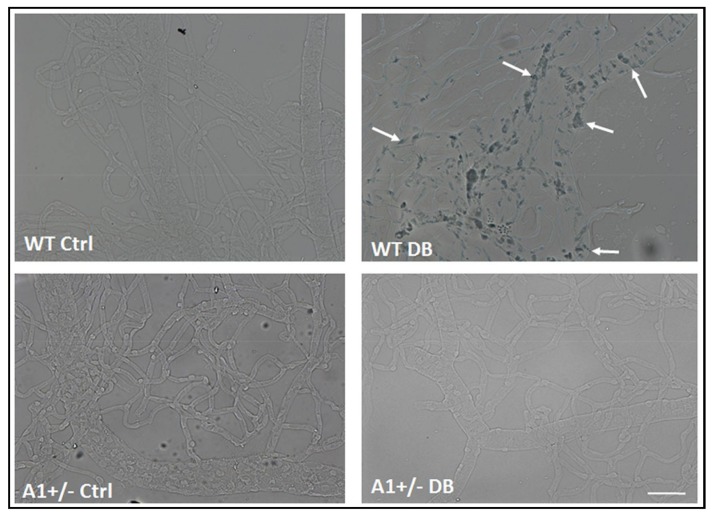
A1 deletion prevents diabetes-induced senescence in retinal vessels. SA β-Gal activity images of isolated retinal vessels showing increased activity (arrows) in vessels isolated from WT diabetic mice. The vessels isolated from A1+/− diabetic retinas are negative for SA β-Gal staining. The non-diabetic control retinas from both WT and A1+/− mice are also negative for SA β-Gal staining. *n* = 3–5. Scale bar = 50 µM.

Analysis of SA β-Gal activity in isolated retinal vessels demonstrated a marked increase in SA β-Gal activity in the vascular cells of the diabetic retinas as compared with those of the non-diabetic controls (Figure 6). The SA β-Gal staining was evident in cells of the larger vessels, as well as in the microvasculature. In order to assess the involvement of arginase 1 expression in this premature senescence phenotype we determined the impact of diabetes on SA β-Gal activity in vessels isolated from arginase 1 heterozygous knockout mice (A1+/−). The vessels from A1+/− diabetic retinas were negative for SA β-Gal staining as were those from non-diabetic control retinas of both WT and A1+/− mice (Figure 6). These results support the role of arginase 1 expression in diabetes-induced premature senescence.

### 2.3. Induction of Endothelial Cell Senescence by High Glucose or Arginase 1 Overexpression and Blockade by Arginase Inhibition

**Figure 7:**
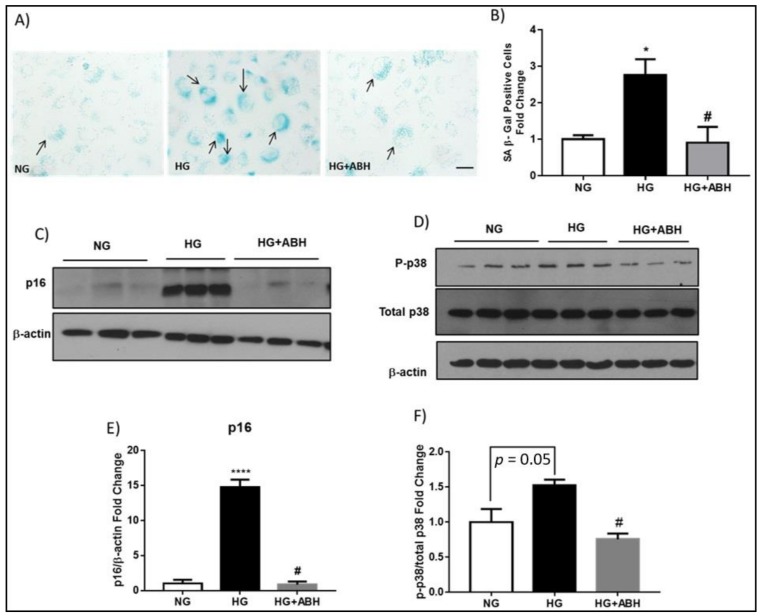
High glucose-induces endothelial cell senescence. (**A**) Senescence associated β-Gal activity (black arrows) and quantification (**B**) showing a significant increase in SA β-Gal positive cells with high glucose (HG, 25 mM) treatment when compared to normal glucose (NG, 5 mM). ABH treatment prevented this increase. * *p* < 0.05 vs. NG. # *p* < 0.05 vs. HG. *n* = 3. Scale bar = 50 µM; (**C**) Western blot analysis with quantification (**E**) showing increased p16^INK4A^ expression in HG treated bovine retinal endothelial cells (BRECs) as compared to NG. Arginase inhibition with ABH blocked this increase. **** *p* < 0.0001 vs. NG. # *p* < 0.0001 vs. HG. *n* = 3. Experiments were repeated at least twice in independent batches of cells. Data are presented as a fold change of the NG; and, (**D**) Western blot analysis with quantification (**F**) showing increased phosphorylation of p38 in HG treated BRECs as compared to NG. Arginase inhibition with ABH significantly blocked this increase. # *p* < 0.05 vs. HG. *n* = 3. Experiments were repeated at least twice in independent batches of cells. Data are presented as a fold change of the NG.

We and others have shown that high glucose induces premature EC senescence [2,17,18]. High glucose treatment also increases expression of arginase 1 in retinal vascular ECs [2,4]. To assess the role of arginase in diabetes/hyperglycemia-induced senescence of retinal microvascular ECs, we determined the effect of high glucose (HG) treatment on senescence of retinal ECs in relation to arginase 1 expression. Treatment of retinal ECs with HG induced upregulation of arginase 1 expression (Supplementary Figure S2) and this was associated with a significant increase in cellular senescence, as demonstrated by increased SA β-Gal activity (Figure 7A,B). Arginase inhibition with ABH prevented this increase and the number of senescent cells was reduced to a level that was comparable to normal glucose NG control conditions. Moreover, the senescence mediator, p16^INK4A^, was significantly increased with HG treatment and this was significantly abrogated with arginase inhibition (Figure 7C,E). High glucose treatment also increased the phosphorylation of the stress marker p38 MAPK, which was also reduced with ABH treatment (Figure 7D,F).

**Figure 8:**
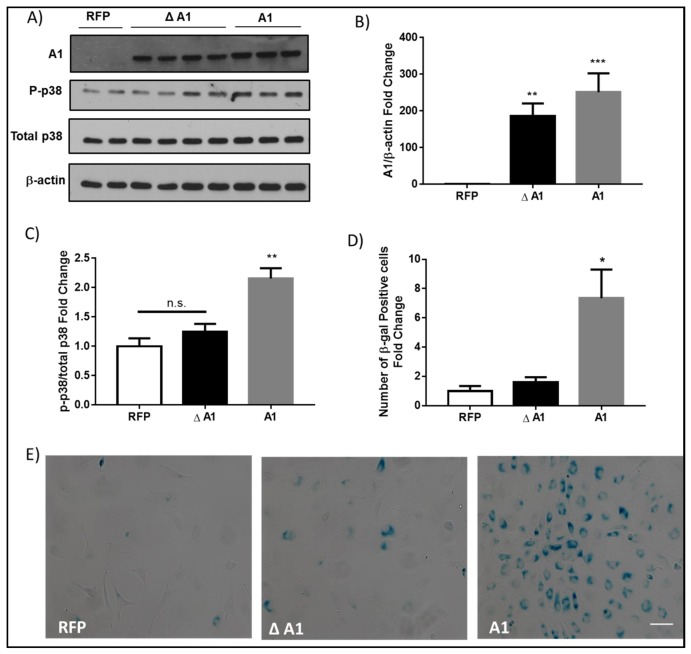
Arginase 1 (A1) overexpression induces endothelial cell senescence. (**A**) Western blot analysis with quantification (**B**) showing levels of A1 protein or (**C**) phosphorylation of the stress marker p38 in bovine retinal endothelial cells (BRECs) transduced with 20 MOI of wild type A1 (A1), inactive mutant A1 (Δ A1), or red fluorescence protein (RFP) adenovirus vectors. The inactive mutant A1 was generated by mutation of aspartic acid 128 to glycine. (**B**) ** *p* < 0.01 vs. RFP. *** *p* < 0.001 vs. RFP. *n* = 6–8. (**C**) n.s., non-significant, ** *p* < 0.01 vs. RFP & Δ A1. *n* = 3–8. Experiments were repeated at least twice in independent batches of cells. Data are presented as a fold change of RFP; (**E**) Senescence associated β-galactosidase (SA β-Gal) activity with quantification (**D**) showing a significant increase in SA β-Gal positive cells with A1 overexpression as compared to RFP or the inactive mutant A1 (Δ A1). * *p* < 0.05 vs. RFP & Δ A1. *n* = 5–10. Experiments were repeated at least twice in independent batches of cells. Data are presented as a fold change of RFP. Scale bar = 50 µM.

To directly determine the impact of increases in arginase 1 expression on endothelial cell senescence we overexpressed active arginase 1 in retinal ECs using adenoviral vector and compared the effects with overexpression of the inactive mutant arginase 1. Studies have shown that mutation of aspartic acid 128 to glycine (D128G) disturbs the binding pocket for manganese, which is a crucial cofactor for arginase activity [19]. When expressed in COS-7 cells, the D128G arginase I mutant was present at the expected molecular weight, but it exhibited a complete loss of activity [20]. Western blot analysis confirmed that arginase 1 protein was significantly increased in retinal ECs that were transduced with either arginase 1 or the D128G inactive mutant arginase 1 (Δ A1) as compared to cells transduced with red fluorescent protein (RFP) adenoviral vector (Figure 8A,B). The overexpression of arginase 1 activated cellular stress mechanisms, as shown by the increased phosphorylation of p38 MAPK (Figure 8A,C) and increased cellular senescence, as shown by the increased numbers of SA β-Gal positive cells (Figure 8D,E) as compared to cells that were transduced with either RFP or Δ A1.

## 3. Discussion

In the current study, we found that a significant increase in arginase 1 protein expression was accompanied by increases in mRNA expression of for the senescence markers p16^INK4A^, p21, p53, and Igfbp3 in diabetic retinas when compared to age matched controls. Analysis of SA β-Gal activity provided further evidence of diabetes-induced premature senescence in both retinal parenchyma and vascular cells. Immunofluorescence analysis of isolated retinal vessels further confirmed the increase in p16^INK4A^ within the vascular endothelial cells. The phosphorylation of p38 MAPK was also increased in the diabetic retina, indicating an increase in cellular stress. Arginase inhibition with ABH significantly blunted all of these alterations. When we challenged the retinal ECs with HG conditions, the number of SA β-Gal positive cells was significantly increased along with increased p16^INK4A^ expression. These effects were substantially reduced with arginase inhibition. Arginase 1 overexpression induced endothelial cell senescence and p38 phosphorylation. This study highlights the role of arginase 1 in inducing EC senescence during diabetic retinopathy.

Arginase has been extensively studied in relation to diabetic complications. Arginase induces vascular dysfunction through competing with eNOS for their common substrate l-arginine, which decreases bioavailable NO and increases oxidative stress [21,22]. Diabetes-induced impairment of endothelial cell derived NO-dependent vasodilation in diabetic rats or mice has been shown to involve increased arginase expression/activity [8,23,24,25]. It has been also found that the upregulation of arginase 1 contributes to decreases in EC-dependent coronary artery dilation in diabetic patients [26]. Arginase inhibition alleviates hypertension associated with type 1 diabetes and restores coronary microvascular function in type 2 diabetes [27,28]. Arginase inhibition also improves microvascular endothelial function in patients with type 2 diabetes [29]. Arginase also is reported to be involved in diabetic nephropathy, as well in impaired corpora cavernosa relaxation in diabetes [30,31,32]. Our group has shown that diabetes induced impairment of retinal blood flow was attenuated in diabetic mice lacking one copy of arginase 1 or diabetic mice treated with arginase inhibitor [5]. We have also reported that arginase 1 expression is increased in the diabetic retina and retinal ECs that were treated with HG [4]. One important mechanism of diabetes-induced A1 expression can be the oxidative stress, which is known to be largely increased under diabetic conditions. Our group has previously shown that oxidative species increase arginase expression/activity in endothelial cells [12]. Overactive arginase contributes to diabetic retinopathy by reducing NO and increasing oxidative stress [2,4]. Thus, the role of overactive arginase in inducing vascular dysfunction in diabetes is well established.

The relationship between arginase expression/activity and p38 MAPK phosphorylation/activation is also well established. Other studies have shown that the inhibition or knockdown of p38 MAPK blocks elevation of arginase 1 expression/activity in conditions of diabetes and angiotensin II-induced endothelial dysfunction [31,33,34]. Our current data showing that the inhibition of arginase prevents diabetes or high glucose-induced increases in phosphorylation of p38 MAPK suggests a feed forward relationship as both processes respond to and create cellular stress. However, the exact mechanisms of how A1 induces p38 MAPK phosphorylation/activation are not fully understood. Reactive oxygen species could serve as key mediators involved in A1-induced activation of p38 MAPK activation under diabetic conditions.

Replicative senescence is the irreversible cell cycle arrest following prolonged cultivation [35]. However, premature senescence occurs in response to cellular stress under conditions of inflammation and oxidative stress and is potentially reversible. In diabetes, the increased formation of advanced glycation end products (AGE) has been found to induce endothelial cell senescence [36]. In human umbilical vein endothelial cells (HUVECs), HG treatment was found to increase the proportion of cells showing increased levels of SA β-Gal activity [37]. Endothelial progenitor cells that were isolated from healthy subjects exposed to HG conditions or endothelial progenitor cells that were isolated from diabetic patients were also found to exhibit enhanced senescence [38]. In the present study, we have shown that A1 induces premature senescence in diabetic retinas. However, the underlying mechanisms of A1-induced premature senescence still need to be explored. Studies using HUVECs have shown that elevation of arginase 1 can induce vascular endothelial inflammation and senescence through eNOS uncoupling [39]. Our group has previously reported that A1 induces endothelial dysfunction through NOS uncoupling and increasing oxidative stress [4,8,40]. We also recently showed that diabetes-induced retinal EC senescence involves sequential events that were initiated by NOX2 activation and production of ROS, leading to increased endothelial arginase expression and activity and decreased production of NO [2]. These reports suggest that decreased NO and increased oxidative stress could be the mechanism of A1-induced endothelial cell senescence in diabetes. Here, we have found that diabetes induced increases in arginase 1 expression promote endothelial cell senescence through a mechanism involving increases in the expression of p16^INK4A^, p21, and p53. 

In the current study, we have shown that arginase inhibition in vivo reversed the diabetic-induced increases in senescence markers. This finding, together with previous reports from our lab and others, emphasize that premature senescence could be reversed or prevented [1,2,41].

Future studies should be conducted to examine a possible role of the arginase 2 isoform in inducing retinal endothelial cell senescence. Overexpression of arginase 2 has been shown to induce senescence in smooth muscle cells and endothelial cells through activating p53/p66Shc signaling pathway [42,43]. To date, there is no isoform-specific arginase inhibitor. In the current study, we used ABH that inhibits both isoforms, so this does not rule out the potential involvement of arginase 2 in inducing retinal endothelial cell senescence in diabetes.

In conclusion, this study shows for the first time that diabetes-induced increases in arginase 1 expression promotes endothelial cell senescence through the activation of p16^INK4A^ and p53 signaling pathways. These results suggest that inhibiting arginase 1 could be a potential therapy for alleviating diabetic retinopathy through decreasing endothelial cell senescence.

## 4. Materials and Methods

### 4.1. Animals and Diabetes Induction

All of the animal procedures performed in this study were conducted, according to the recommendations in the National Institutes of Health Guide for the Care and Use of Laboratory Animals. The Animal Protocol was approved by the Institutional Animal Care and Use Committee of Augusta University. Homozygous deletion of A1 is postnatally lethal, so we used heterozygous arginase 1 knockout (A1+/−) mice (B6.129-Arg1tm1Rki/J, Jackson Labs, Bar Harbor, ME, USA, stock# 007741). Male littermate wild type (WT) mice were used as controls for A1 knockout studies. WT (C57BL/6J) mice from our in-house colony were used for arginase inhibition studies. Seven to eight-week-old male mice were rendered diabetic by intraperitoneal injection (IP) of streptozotocin (STZ, 60 mg/kg) (Sigma-Aldrich, St. Louis, MO, USA; Cat. # S0130) that was dissolved in sodium citrate buffer (0.1 M, pH 4.5) on five successive days. Mice were screened for diabetes beginning five days after the first dose of STZ by assessing urine glucose levels with urine testing strips. Mice with fasting urine glucose levels higher than 500 mg/dL were considered to be diabetic. Studies were performed after 8–24 weeks of diabetes. Some groups of mice were treated with the arginase inhibitor 2(S)-Amino-6-boronohexanoic acid (ABH, 10 mg/kg/day, IP) or vehicle (saline). The ABH treatment was started at six weeks after the onset of diabetes and continued for two weeks. Total number of mice used for the current study was 235.

### 4.2. Retinal Vessels Isolation

Retinal vessels were isolated by two different methods depending on the outcome measure. For PCR and β-gal staining, fresh retinas were dissected and placed in water on ice for 1 h, followed by treatment with deoxyribonuclease I (116 U/mL, 25–30 min, Worthington Biochemical Corp., Lakewood, NJ, USA; Cat. # LS006361), as described previously [4]. Retinal vessels were then rinsed to remove contaminating neurons and glia and processed for PCR array or quantitative RT-PCR. For PCR array and RT-PCR, each sample contained six retinal vessel nets from six animals of the same experimental group. For β-gal activity assays, vessels were flat mounted on silane-coated slides and processed for SA β-gal activity assay.

For p16 staining, Retinas were carefully dissected out from 4% paraformaldehyde fixed eyeballs and retinal vessels were isolated using trypsin digestion method, as previously described [9]. The isolated retinal vasculature was then snap frozen in optimal cutting temperature (OCT) solution.

### 4.3. Cell Culture Studies

Bovine retinal endothelial cells (BRECs) were isolated as previously reported [44]. Passages 5–9 were used for experiments. BRECs were maintained in complete medium containing M199 (Gibco Thermo Fisher, Waltham, MA, USA), 10% fetal bovine serum (FBS), Penicillin/Streptomycin (Gemini, West Sacramento, CA, USA), and 10% complete medium from Cell Systems until they reached 70–80% confluency. Cells were then shifted to M199 medium containing 0.2% FBS, 0.1% bovine serum albumin (BSA), 50 µM l-Arginine, and 5.5 mM d-glucose (normal glucose, NG) or 25 mM d-glucose (high glucose, HG) for 3–5 days. Some cells were treated with arginase inhibitor 2(S)-amino-6-boronohexanoic Acid (ABH, 100 µM) together with HG or NG. Cells were then harvested and processed for western blotting, as previously described [4].

For studies of the effects of arginase upregulation on premature EC senescence, BREC were grown to 70–80% confluency and transduced, as previously described, to overexpress arginase 1 using adenoviral vectors [20]. Cells were treated with adenoviral vector carrying wild type arginase 1 (A1) or inactive mutant (Δ A1) at 20 multiple of infection (MOI) in low arginine media containing 0.2% FBS and 0.1% BSA for 6 h. The MOI was chosen according to the results of preliminary studies. Red fluorescence protein (RFP) adenoviral vector was used as control to visualize the transduced cells. Media was then changed to fresh media contacting 0.2% FBS and 0.1% BSA for 24 h. Cells were then harvested for western blotting or fixed for SA β-gal enzyme activity assay.

### 4.4. PCR Analyses

A PCR array was used to examine senescence-related mRNA expression. Total RNA was extracted from the fresh retinal vessels using RNAqueous™-4PCR kit (Invitrogen, Carlsbad, CA, USA; Cat. # AM1914) with minor modifications. Equal amounts of total RNA (250 ng) from each sample were reverse-transcribed into cDNA using the RT First Strand Kit (Qiagen Inc., Germantown, MD, USA; Cat. # 330401), according to the manufacturer’s instructions. Quantitative PCR was performed following the manufacturer’s protocol using Mouse Cellular Senescence RT2 Profiler PCR Array (Qiagen Inc., Germantown, MD, USA; Cat. # 330231 PAMM-050ZA) containing primers of 84 testing genes that are involved in the cellular senescence process. β-actin was used as the internal control gene. Fold changes of mRNA abundance were calculated with CT values using the ΔΔCT method. Fold regulation was calculated according to Qiagen data analysis center web tool. For all fold-change values that are greater than 1, the fold-regulation and fold-change values are the same. For all fold-change values less than 1, the fold regulation is the negative inverse of the fold change.

Results of the PCR Array analyses were confirmed by further studies using quantitative RT-PCR. Total RNA was extracted and reverse-transcribed, as previously described [9]. Quantitative PCR was performed using an ABI StepOne Plus Thermocycler (Applied Biosystems, Foster City, CA, USA) with TaqMan gene expression assays (Invitrogen, Carlsbad, CA, USA) or master mix (Power SYBR Green; Applied Biosystems, Foster City, CA, USA). TaqMan assay probes used to detect Cdkn2a (p16), Cdkn1a (p21), Insulin-like growth factor-binding protein 3 (Igfbp-3), and hypoxanthine phosphoribosyltransferase (HPRT) as internal control, were Mm00494449_m1, Mm04205640_g1, Mm01187817_m1 and Mm00446968_m1. Primer sequences for mouse transcripts were as follows: p53 For-5′-GAT ATC AGC CTC GAG CTC CC-3′; p53 Rev-5′-TCC ATG CAG TGA GGT GAT GG-3′; HPRT For-5′-GAA AGA CTT GCT CGA GAT GTC ATG-3′; HPRT Rev-5′-CAC ACA GAG GGC CAC AAT GT-3′. Data were normalized to HPRT and the fold change between levels of different transcripts was calculated by the ΔΔC_T_ method.

### 4.5. Western Blot

Protein lysates from cells and animal tissues were prepared, as previously described [9]. Briefly, retinal protein extracts were prepared using RIPA buffer (Millipore, Billerica, MA, USA) containing 1× protease and phosphatase inhibitors (Complete Mini and phosSTOP, respectively; Roche Applied Science, Indianapolis, IN, USA). Proteins were separated on SDS-PAGE and were then transferred to nitrocellulose membrane (Millipore, Billerica, MA) and blocked in 5% milk (Bio-Rad, Hercules, CA, USA) for one hour. Different primary antibodies were incubated overnight at 4 °C. The primary antibodies used were arginase 1 (Santa Cruz Biotechnology, Dallas, TX, USA; Cat. # Sc-20151; 1:500), p16^INK4A^ (Abcam, Cambridge, MA, USA; Cat. # ab189034; 1:500) phospho-p38 (Cell Signaling Technology, Danvers, MA, USA; Cat. # 4511; 1:500), total p38 (Cell Signaling Technology, Cat. # 9212, 1:500), tubulin (Sigma-Aldrich, Cat. # T-9026, 1:5000), and β-actin (Sigma-Aldrich, Cat. # A1978, 1:5000). The next day, membranes were washed in TBST (Tris-buffered saline with 0.5% Tween-20) and horseradish peroxidase-conjugated secondary antibodies (GE Healthcare, Piscataway, NJ, USA) were added (1:5000 for tubulin and actin and 1:1000 for others). Enhanced chemiluminescence system (GE Healthcare Bio-Science Corp., Piscataway, NJ, USA) was used to detect immunoreactive proteins. Data were quantified by densitometry using ImageJ and normalized to loading control.

### 4.6. SA β-Galactosidase Activity

Activity of the senescence associated β-galactosidase enzyme (SA β-Gal) has been recognized as a valid standard for detecting senescent cells [16]. Activity of SA β-Gal was measured in both retinal sections and isolated vessels, as previously described using the Senescence Detection Kit (Bio Vision laboratory, Milpitas, CA, USA; Cat. # K320) [2].

BRE cells were grown on sterile coverslips and subjected to HG insult or A1 overexpression. At the end of the experiment, the cells were fixed according to manufacturer’s instructions (Bio Vision laboratory, Milpitas, CA, USA) then incubated overnight in the staining solution at 37 °C.

Carl Zeiss Anxioplan2 fluorescence microscope was used for taking bright field images. At least ten images per BRECs samples were taken randomly. Then, the number of SA β-Gal positive cells were counted using Image J software in each sample and normalized to controls.

### 4.7. Immunofluorescence Analysis of Isolated Retinal Vessels

The isolated retinal vasculature was snap frozen in optimal cutting temperature (OCT) solution. Cryostat sections (10 μm) were obtained, mounted on glass slides and permeabilized with 1% Triton for 10 min and blocked in 10% normal goat serum for 1 h. Sections were then incubated with anti-p16^INK4A^ antibody (Abcam, Cambridge, MA, USA; Cat. # ab189034; 1:50) and Alexa Fluor 594-conjugated isolectin GS-IB4 (Invitrogen, Carlsbad, CA, USA; Cat. # I21413; 1:200) at 4 °C overnight. On the next day, the sections were washed three times with phosphate-buffered saline (PBS) and were then incubated for 1 h at room temperature with Alexa Fluor 488-conjugated secondary antibody (Invitrogen, Carlsbad, CA, USA; Cat. # A11034; 1:400) that was washed in PBS, and covered with mounting medium containing 4′,6′-diamino-2-phenylindole (DAPI) for counterstaining (Vectashield; Vector Laboratories, Burlingame, CA, USA; Cat. # H-1200). Images from three to four samples per group were taken using Zeiss Axioplan2 Imager Microscope (Zeiss, San Diego, CA, USA). The results were analyzed by measuring the fluorescence intensity of p16 immunolabeling using ImageJ software.

### 4.8. Statistical Analysis

GraphPad Prism 7 (GraphPad Softwar Inc., La Jolla, CA, USA) was used for statistical analysis. One Way ANOVA followed by Bonferroni test was used for multiple comparisons. The student’s *t*-test (Two-tailed) was used in cases of single comparisons. *p* ≤ 0.05 was considered to be statistically significant. For in vitro studies, experiments were repeated at least twice in independent batches of cells. Results were presented as mean ± SEM. For PCR array results, differentially regulated genes were defined based on a fold cut-off of 1.5 and *p* value cut-off of <0.05 that was calculated using the Student *t*-test. For PCR analyses of isolated retinal vessels, vessels from six retinas were pooled and the analysis was repeated for at least three sets of pooled retinas. For in vitro studies, experiments were repeated at least twice in independent batches of cells.

## Supplementary Materials

Supplementary materials can be found at http://www.mdpi.com/1422-0067/19/4/1215/s1.

## Abbreviations

A1	Arginase1
A1+/−	A1 heterozygous knockout
A2	Arginase 2
ABH	[2(S)-Amino-6-boronohexanoic acid], arginase inhibitor
AGE	Advanced glycation end products
BRECs	Bovine retinal endothelial cells
BSA	Bovine serum albumin
DAPI	4′,6′-diamino-2-phenylindole
ECs	Endothelial cells
FBS	Fetal bovine serum
HG	High glucose
HPRT	Hypoxanthine phosphoribosyl transferase
HUVECs	Human umbilical vein endothelial cells
Igfbp3	Insulin-like growth factor binding protein-3
MOI	Multiple of infections
NG	Normal glucose
NO	Nitric oxide
NOS	Nitric oxide synthase
NOX2	NADPH oxidase 2
OCT	Optimal cutting temperature
P16^INK4a^	cyclin-dependent kinase inhibitor 2A
P21	cyclin-dependent kinase inhibitor 1
P53	Tumor suppressor p53
PBS	Phosphate-buffered Saline
RFP	Red fluorescence protein
SA β-Gal	Senescence associated β-galactosidase
STZ	Streptozotocin
WT	Wild type
WT Ctrl	Wild type control
WT DB	Wild type diabetic
Δ A1	Inactive mutant arginase 1
