# Supplementary material for: Mechanisms of Diabetes-Induced Endothelial Cell Senescence: Role of Arginase 1
Source: Int J Mol Sci. 2018 Apr 17;19(4):1215. doi: 10.3390/ijms19041215 (PMC5979610; doi:10.3390/ijms19041215)
Supplement: Supplementary file 1 [file ijms-19-01215-s001.pdf]

Supplementary Table S1. PCR array analysis. Results from RT2 Profiler Mouse Cellular Senescence, cut-off of 1.-fold regulation and p < 0.05) show upregulation of Cdkn1a, Ifgbp3, and Igfbp7.

Gene Symbol	Description	Fold Regulation	*p* Value
Abl1	C-abl oncogene 1, non-receptor tyrosine kinase	−1.06	0.57
Akt1	Thymoma viral proto-oncogene 1	1.02	0.57
Aldh1a3	Aldehyde dehydrogenase family 1, subfamily A3	1.22	0.31
Atm	Ataxia telangiectasia mutated homolog (human)	−1.33	0.20
Bmi1	Bmi1 polycomb ring finger oncogene	−1.16	0.36
Calr	Calreticulin	−1.43	0.01
Ccna2	Cyclin A2	−1.26	0.10
Ccnb1	Cyclin B1	1.18	1.00
Ccnd1	Cyclin D1	−1.01	0.91
Ccne1	Cyclin E1	−1.27	0.02
Cd44	CD44 antigen	1.01	1.00
Cdc25c	Cell division cycle 25 homolog C (S. pombe)	−1.39	0.17
Cdk2	Cyclin-dependent kinase 2	1.15	0.39
Cdk4	Cyclin-dependent kinase 4	1.06	0.50
Cdk6	Cyclin-dependent kinase 6	1.03	0.78
**Cdkn1a**	**Cyclin-dependent kinase inhibitor 1A (P21)**	**2.83**	**0.01**
Cdkn1b	Cyclin-dependent kinase inhibitor 1B	−1.03	0.69
Cdkn1c	Cyclin-dependent kinase inhibitor 1C (P57)	1.45	0.19
Cdkn2a	Cyclin-dependent kinase inhibitor 2A	1.46	0.26
Cdkn2b	Cyclin-dependent kinase inhibitor 2B (p15)	−1.20	0.35
Cdkn2c	Cyclin-dependent kinase inhibitor 2C (p18)	−1.39	0.0018
Cdkn2d	Cyclin-dependent kinase inhibitor 2D (p19)	−1.20	0.12
Chek1	Checkpoint kinase 1 homolog (S. pombe)	−1.30	0.23
Chek2	CHK2 checkpoint homolog (S. pombe)	1.67	0.25
Cited2	Cbp/p300-interacting transactivator	−1.08	0.42
Col1a1	Collagen, type I, alpha 1	1.38	0.17
Col3a1	Collagen, type III, alpha 1	−1.14	0.74
Creg1	Cellular repressor of E1A-stimulated genes 1	−1.20	0.20
E2f1	E2F transcription factor 1	1.10	0.96
E2f3	E2F transcription factor 3	−1.01	0.89
Egr1	Early growth response 1	1.12	0.98
Ets1	E26 avian leukemia oncogene 1, 5' domain	1.24	0.17
Ets2	E26 avian leukemia oncogene 2, 3' domain	−1.06	0.65
Fn1	Fibronectin 1	1.30	0.0021
Gadd45a	Growth arrest and DNA-damage-inducible 45 α	1.00	0.95
Glb1	Galactosidase, beta 1	−1.15	0.44
Gsk3b	Glycogen synthase kinase 3 beta	−1.14	0.15
Hras1	Harvey rat sarcoma virus oncogene 1	−1.19	0.31
Id1	Inhibitor of DNA binding 1	1.44	0.01
Ifng	Interferon gamma	undetected	
Igf1	Insulin-like growth factor 1	−1.02	0.80
Igf1r	Insulin-like growth factor I receptor	−1.08	0.26

**Igfbp3**	**Insulin-like growth factor binding protein 3**	**8.13**	**0.01**
Igfbp5	Insulin-like growth factor binding protein 5	1.10	0.71
**Igfbp7**	**Insulin-like growth factor binding protein 7**	**1.63**	**0.02**
Ing1	Inhibitor of growth family, member 1	1.07	0.69
Irf3	Interferon regulatory factor 3	−1.04	0.75
Irf5	Interferon regulatory factor 5	1.03	0.74
Irf7	Interferon regulatory factor 7	1.98	0.06
Map2k1	Mitogen-activated protein kinase kinase 1	−1.22	0.14
Map2k3	Mitogen-activated protein kinase kinase 3	1.06	0.73
Map2k6	Mitogen-activated protein kinase kinase 6	−1.01	0.88
Mapk14	Mitogen-activated protein kinase 14	−1.02	0.87
Mdm2	Transformed mouse 3T3 cell double minute 2	−1.05	0.66
Morc3	Microrchidia 3	−1.09	0.65
Myc	Myelocytomatosis oncogene	1.24	0.69
Nbn	Nibrin	1.02	0.98
Nfkb1	Nuclear factor of kappa light polypeptide gene enhancer in B-cells 1, p105	1.12	0.15
Nox4	NADPH oxidase 4	1.19	0.74
Pcna	Proliferating cell nuclear antigen	−1.10	0.34
Pik3ca	Phosphatidylinositol 3-kinase, catalytic, alpha polypeptide	−1.09	0.21
Plau	Plasminogen activator, urokinase	1.45	0.55
Prkcd	Protein kinase C, delta	1.11	0.13
Pten	Phosphatase and tensin homolog	−1.23	0.15
Rb1	Retinoblastoma 1	−1.09	0.43
Rbl1	Retinoblastoma-like 1 (p107)	1.06	0.69
Rbl2	Retinoblastoma-like 2	−1.15	0.25
Serpinb2	Serine (or cysteine) peptidase inhibitor, clade B, member 2	undetected	
Serpine1	Serine (or cysteine) peptidase inhibitor, clade E, member 1	−1.30	0.32
Sirt1	Sirtuin 1 (silent mating type information regulation 2, homolog) 1 (S. cerevisiae)	−1.10	0.35
Sod1	Superoxide dismutase 1, soluble	1.07	0.88
Sod2	Superoxide dismutase 2, mitochondrial	−1.09	0.55
Sparc	Secreted acidic cysteine rich glycoprotein	1.18	0.18
Tbx2	T-box 2	−1.05	0.47
Tbx3	T-box 3	−1.10	0.66
Terf2	Telomeric repeat binding factor 2	−1.08	0.48
Tert	Telomerase reverse transcriptase	−1.19	0.53
Tgfb1	Transforming growth factor, beta 1	1.40	0.08
Tgfb1i1	Transforming growth factor beta 1 induced transcript 1	1.45	0.0020
Thbs1	Thrombospondin 1	1.03	0.84
Trp53	Transformation related protein 53	1.09	0.67
Trp53bp1	Transformation related protein 53 binding protein 1	−1.08	0.67
Twist1	Twist homolog 1 (Drosophila)	1.69	0.27
Vim	Vimentin	1.09	0.54

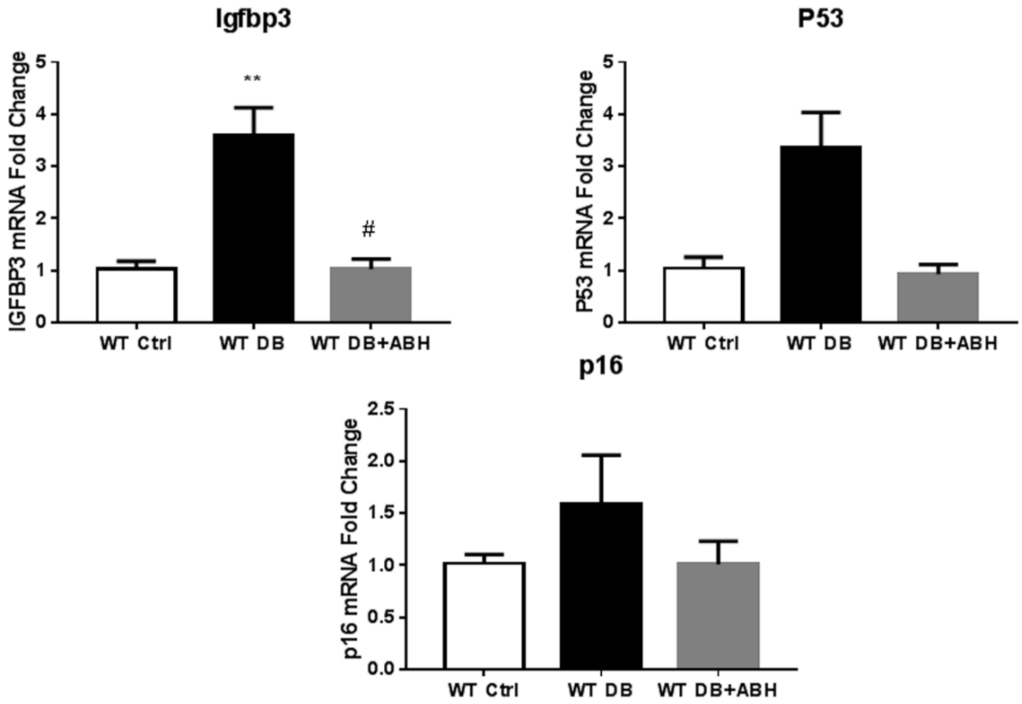

Supplementary Figure S1. Arginase inhibition prevented diabetes induced senescence in isolated retinal vessels. qRT-PCR showing a significant increase of Igfbp3 in vessels isolated from the diabetic retinas along with a trend towards increases in p16 and p53. ABH treatment prevented these alterations ** $p < 0.01$ vs. WT Ctrl. # $p < 0.05$ vs. WT DB. $n = 3–12$.

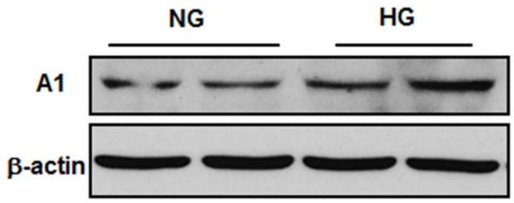

Supplementary Figure S2. High glucose induced arginase 1 expression in endothelial cells. Western blot analysis showing increased A1 expression in bovine retinal endothelial cells (BRECs) treated with high glucose (HG, 25 mM) compared to normal glucose (NG, 5mM).
